# Subjective Size Perception Depends on Central Visual Cortical Magnification in Human V1

**DOI:** 10.1371/journal.pone.0060550

**Published:** 2013-03-25

**Authors:** D. Samuel Schwarzkopf, Geraint Rees

**Affiliations:** 1 Wellcome Trust Centre for Neuroimaging, University College London, London, United Kingdom; 2 Institute of Cognitive Neuroscience, University College London, London, United Kingdom; 3 Cognitive Perceptual & Brain Sciences, University College London, London, United Kingdom; Cardiff University, United Kingdom

## Abstract

In the Ebbinghaus illusion, the context surrounding an object modulates its subjectively perceived size. Previous work implicates human primary visual cortex (V1) as the neural substrate mediating this contextual effect. Here we studied in healthy adult humans how two different types of context (large or small inducers) in this illusion affected size perception by comparing each to a reference stimulus without any context. We found that individual differences in the magnitudes of the illusion produced by either type of context were correlated with V1 area defined through retinotopic mapping using functional MRI. However, participants' objective ability to discriminate the size of objects presented in isolation was unrelated to illusion strength and did not correlate with V1 area. Control analyses showed no correlations between behavioral measures and the overall V1 area estimated probabilistically on the basis of neuroanatomy alone. Therefore, subjective size perception correlated with variability in central cortical magnification rather than the anatomical extent of primary visual cortex. We propose that such changes in subjective perception of size are mediated by mechanisms that scale with the extent to which an individual's V1 selectively represents the central visual field.

## Introduction

**Figure 1:**
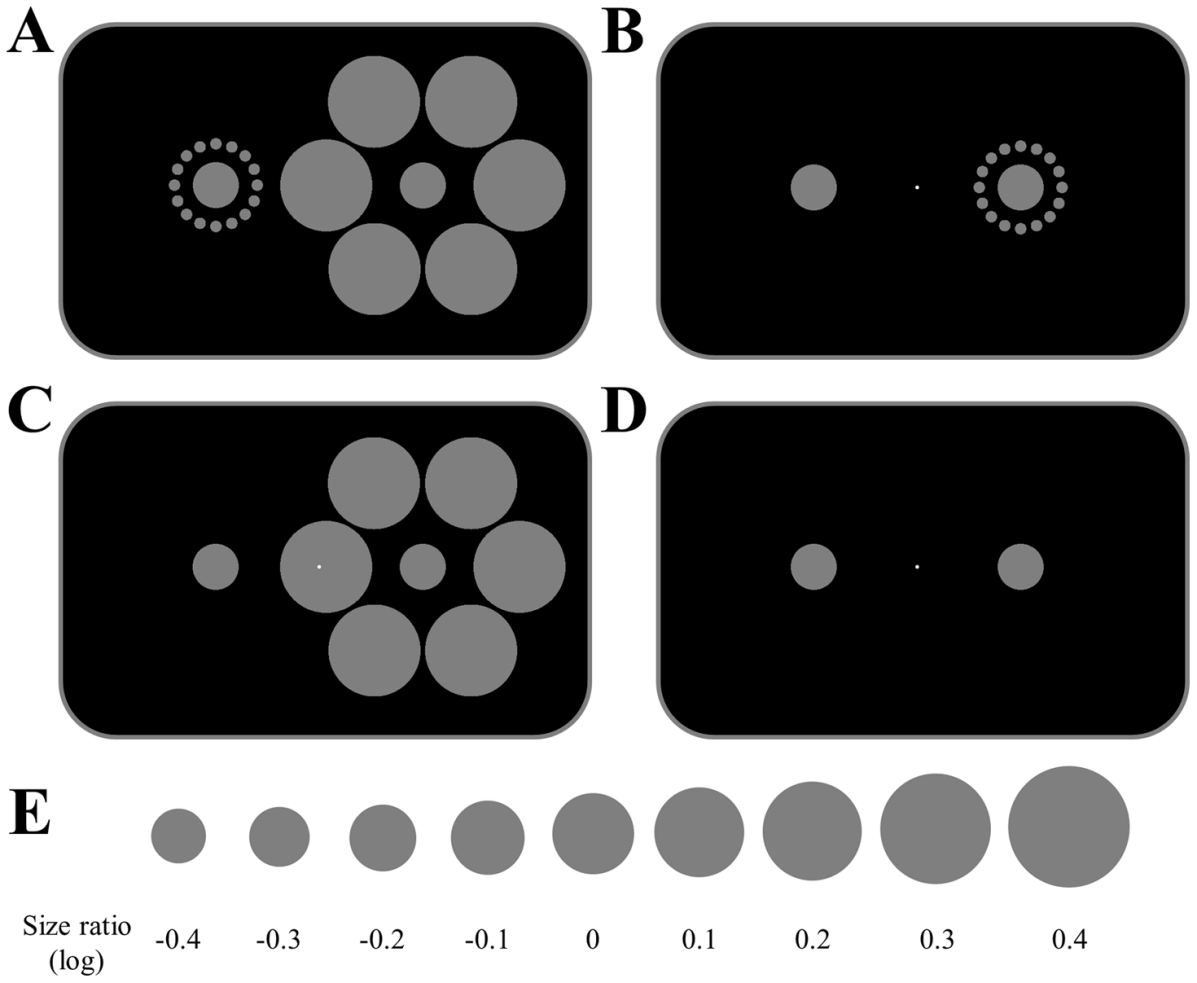
The Ebbinghaus illusion. A. In the classical form of the illusion two identical circles are surrounded by smaller (left) or larger (right) inducers. This causes a perceived difference in the size of the two central circles. B–D. Example stimuli (all without any physical size difference between test and reference) for the three stimulus conditions. Participants fixated the small white dot while two circles were shown to the left or right of fixation. One circle (the left in all these examples) was the reference and always remained constant. The other circle was the test stimulus and could either be surrounded by small inducers (B), large inducers (C), or no inducers (D). The hemifield where the test stimulus appeared was pseudo-randomized and counterbalanced for each participant. E. The size of the test stimulus varied between 9 different test/reference size ratios on a logarithmic scale (shown here schematically).

Visual illusions allow us to study the neural mechanisms associated with our subjective experience of the world, because they dissociate the perceived quality of an image from its physical representation. In the classical Ebbinghaus illusion (Figure 1A), two identical targets are surrounded by a circular arrangement of inducers that are either smaller or larger, respectively, than the targets [1]. This results in a perceived difference in the size of the targets, so that the one surrounded by small inducers appears larger than the one surrounded by large inducers.

Previous behavioral and neuroimaging work suggests that local circuits in human primary visual cortex (V1) may mediate such illusory size perception. For instance, the illusion only displays partial interocular transfer; it is reduced when the inducers and target stimuli are presented to different eyes [2]. This is a hallmark of effects mediated in V1 because this is the first area along the visual processing pathway where information from both eyes is combined, but a large proportion of neurons are still monocular [3]. Moreover, the spatial extent of V1 activation measured using functional MRI reflects the perceived size of an object [4]–[6]. Furthermore, Ebbinghaus illusion strength is negatively correlated with the surface area of V1, consistent with the notion that the spatial spread of neuronal connections between the target and inducers mediates the effect, which is thus weaker in larger cortices [7]. Finally, the perceived size of retinal afterimages is modulated by illusory size perception [8] suggesting that even though activation is kept constant on the retina more central processes are involved in creating subjective experience of stimulus size.

However, these findings leave a number of questions unresolved. First, it remains unclear which exact mechanisms mediate the changes in perceived size in the Ebbinghaus illusion. Psychophysical experiments indicate that under most stimulus conditions both small and large inducers produce a *reduction* in perceived size [9]. This runs counter to the common intuition that the target surrounded by smaller inducers generally appears larger. Second, in our earlier experiments we observed a significant hemispheric asymmetry such that the correlation between V1 surface area and Ebbinghaus strength was specific to left V1 [7]. This could indicate a particular hemispheric bias for processing fine spatial detail or because participants only use one visual hemifield for their illusion judgments, even though a stimulus was presented in each hemifield in that earlier experiment. Third, it is unknown whether objective size discrimination, as opposed to subjective judgments of the illusory difference, is also related to V1 area. If that were the case, the relationship between illusion strength and V1 could merely be an epiphenomenon of differences in participants' ability to perform the visual discrimination task. Local V1 area (cortical magnification) is correlated with individuals' Vernier acuity [10], that is, the ability to discriminate very fine visual detail – arguably a function also related to making fine judgments of the size of two objects. Fourth, because we defined V1 functionally through retinotopic mapping in our earlier work, it is not clear whether the anatomically defined extent of V1 or rather the variability in cortical magnification is relevant for illusion strength.

To address these issues we measured the strength of the Ebbinghaus illusion separately for the two different contexts, that is, targets surrounded, respectively, by large or small inducers. Participants were asked to judge whether the target inside the inducers presented to one visual hemifield was larger or smaller than a fixed-size reference stimulus without any inducers presented to the opposite hemifield (Figure 1B–D). In the same individuals we measured the surface area of early visual areas V1–V3 using standard retinotopic mapping procedures [11]. This allowed us to test the direction of the illusory effects separately for each context, their relationship with V1 surface area, and for any potential hemispheric asymmetry by presenting the illusion stimulus systematically in different visual hemifields.

## Materials and Methods

### Participants

Twenty-six normal, healthy, human volunteers (11 female, 5 left-handed, age range: 19–36) all with normal or corrected-to-normal visual acuity participated in this experiment. Participants gave written informed consent and all procedures were approved by the UCL Research Ethics Committee.

### Procedure

Participants took part in two independent experimental sessions. In a behavioral session they completed two runs of the psychophysical measurements to measure the strength of the Ebbinghaus illusion stimuli plus objective size discrimination performance. In a second imaging session, functional magnetic resonance imaging (fMRI) was used to delineate the location and surface area of early visual cortical areas in each individual. The two sessions were conducted under different external conditions as they took place in different locations (inside the MRI scanner vs a psychophysical testing room) and were usually separated by several days or, in some cases, months.

### Psychophysical experiments

Participants were seated in a darkened room in front of a computer monitor. They placed their head in a forehead-and-chin rest to maintain a constant viewing distance of 57 cm. In each experimental trial they viewed the illusion stimuli displayed on the computer screen and keyed their response by pressing one of two buttons on the computer keyboard. Stimuli were generated in Psychtoolbox 3 [12] running under the MATLAB (MathWorks Inc.) programming environment using custom scripts.

Participants were instructed to maintain fixation on a small white dot (diameter: 0.16°, luminance: 164 cd/m^2^) in the center of the screen at all times. Every 54 trials participants were given a break to allow them to relax their eyes. Each trial block was started by a button press from the participant. A trial started with a short fixation period (500 ms) in which only the fixation dot was displayed against a dark gray background (luminance: 0.18 cd/m^2^). This was followed by a 100 ms interval in which two stimuli were shown, one to the left and one to the right of fixation (the center of each stimulus was located at 4.65° eccentricity). One of the stimuli, the reference, was a filled circle (luminance: 41 cd/m^2^) which was always fixed in size (diameter: 1.03°). The other stimulus could be one of three different conditions: the Ebbinghaus context with small inducers (Figure 1B, the context with large inducers (Figure 1C), or a control stimulus that only contained the target circle but no inducers (Figure 1D). The stimulus with small inducers comprised 16 filled circles (diameter: 0.26°) that were located 1.86° from the center of the target. The stimulus with large inducers comprised 6 circles (diameter: 2.07°) separated 4.34° from the center of the target.

Subsequently, the stimulus and the fixation dot disappeared and participants made their response. This behavioral response then triggered the start of the next trial. In separate runs they were to indicate on which side of fixation the target was either larger or smaller to counteract any potential confounding effects of response bias. The order of these runs was counterbalanced across participants. There were 972 trials in each run.

On different trials we varied the diameter of the target inside the illusion (or the control stimulus) relative to the diameter of the reference. There were 9 different size ratios: 0.67–1.49, in nine equal logarithmic steps (Figure 1E). The illusion stimuli could either be presented to the left or right visual hemifield while the constant reference would be shown to the opposite hemifield. There were 18 trials for each combination of size ratio, contextual condition, and hemifield. The order of these trial types was pseudo-randomized but counterbalanced within each trial block.

To quantify the illusion strength (or size discrimination performance, in the case of the control condition) we plotted the proportion of trials when each participant responded that the test stimulus was larger than the reference against the size ratio of the stimuli (test relative to reference, on a logarithmic scale). We used a maximum likelihood procedure [13] to fit a cumulative Gaussian psychometric function to these data. Illusion strength (or bias in the control stimulus) was quantified as the point of subjective equality (PSE), that is, the threshold size ratio at which the participant perceived the two target stimuli to be equally large. Further, we used the slope of these psychometric curves to estimate each participant's sensitivity in the task.

For group level comparisons we conducted curve fitting on data pooled regardless of which hemifield the illusion stimulus was presented in. Unless specified, for analyses of individual differences we performed this curve fitting separately for trials in which the illusion (or variable control) stimuli were presented to the left or right visual hemifield, respectively, which afforded two data points for each participant. We tested individual differences through Spearman's *rho* rank correlation and bootstrapping the 95% confidence interval by resampling the data 10000 times with replacement. If this confidence interval differs notably from the nominal confidence interval for a given correlation coefficient and sample size, this is an indication that distributional assumptions of the statistical test may not hold. Therefore, in rare situations where the absolute difference between the confidence intervals (summed for upper and lower bounds) exceeded 0.05, we further calculated Shepherd's *pi* correlation, i.e. Spearman's rho after removing potential bivariate outliers identified through the bootstrapped Mahalanobis distance and adjusting the p-value [14]. This approach is more robust whilst not unduly sacrificing statistical power as is the case when applying robust statistics by default.

### Retinotopic mapping

Procedures for measuring the surface area of V1 and related visual areas have been described previously, and 17 of the present participants had already participated in that earlier study [7]. Briefly, participants lay inside the bore of a Siemens TIM Trio 3T MRI scanner and viewed visual stimuli projected on a screen at the back of the bore by means of a front-surface mirror attached to the head coil. We used rotating wedge and expanding/contracting ring stimuli containing a flickering checkerboard pattern to map retinotopic responses in visual cortex. Ring stimuli were only used in a subset of participants. Data were preprocessed in SPM8 (http://www.fil.ion.ucl.ac.uk/spm) and the time series were analyzed using Fourier transform to extract the phase and power at the fundamental frequency of the stimulus. 3D reconstructions of the boundary between grey and white matter were generated and inflated in Freesurfer [15], [16]. Retinotopic maps were then projected onto the inflated cortical surface for each participant and the boundaries of V1 delineated manually by taking into account the reversals in the polar map and the peripheral extent of the significant (p<0.05) activation to the polar mapping stimulus (F-test based on dividing power at fundamental frequency by those of all other frequencies). Eccentricity maps, when present (18 participants), were used to inspect these borders but were not used to determine the peripheral edge of V1 because eccentricity estimates from phase-encoded methods can be subject to bias [17].

## Results

### Illusion measurements

**Figure 2:**
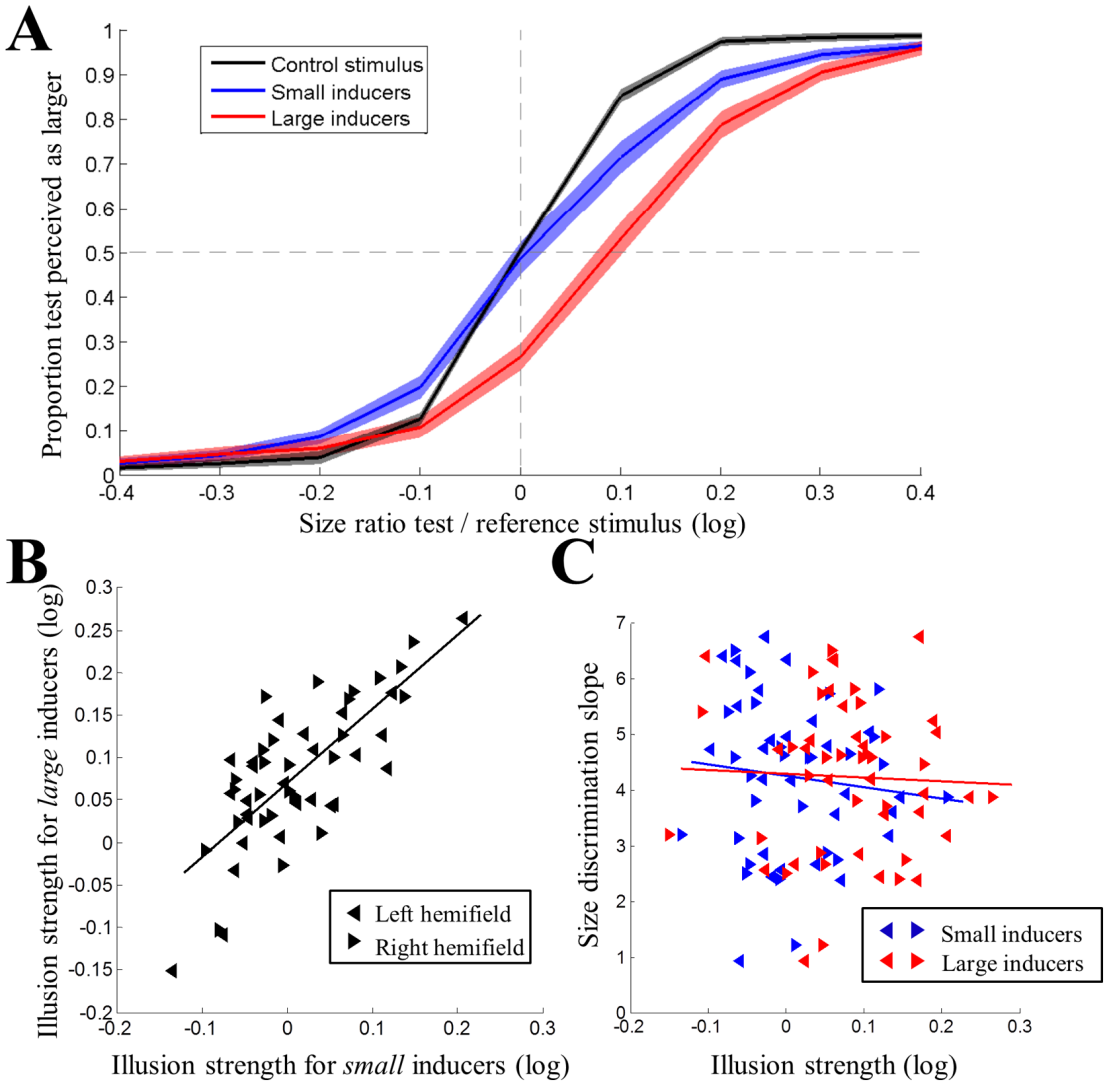
Behavioural measurement of Ebbinghaus illusion. A. Group-level psychometric curves for the three stimulus conditions. The proportion of trials the test was perceived as larger is plotted against the test/reference size ratio (in logarithmic units). Shaded regions denote ±1 standard error of the mean across participants. A rightward shift of the curve suggests the point of subjective equality is positive; thus the illusion is a reduction in perceived size of the target. Black: control stimuli. Blue: small inducers. Red: large inducers. B. Individual illusion strengths for large inducers plotted against those for small inducers. C. Individual size discrimination slopes plotted against illusion strengths. Blue: small inducers. Red: large inducers. In both B and C, left and right pointing triangles denote measurements from left and right visual hemifields in individual participants, respectively. Solid lines are linear regression fits.

In behavioral experiments in 26 participants we measured the psychometric curves for judging the size of three different stimulus configurations relative to a fixed-size reference: the Ebbinghaus illusion with large inducers, the Ebbinghaus illusion with small inducers, and a control stimulus without any inducers. Figure 2A shows psychometric curves averaged across participants plotting the proportion of trials the test stimulus was perceived to be larger than the reference against the actual size ratio of the two target stimuli (test stimulus relative to reference, in logarithmic units).

However, there were significant differences in the biases between the three stimulus conditions (repeated-measures ANOVA; F(2,50) =  = 29.51, p<0.0001). On average the illusion strength (point of subjective equality) for large inducers was significantly greater than for small inducers (paired t-test; t(25) = 8.34, p<0.0001, Cohen's d = 1.64) and the bias for the control stimulus (t(25) = 6.17, p<0.0001, d = 1.21). There was no difference between the illusion strength for small inducers and the control stimulus (t(25) = 1.22, p = 0.233, d = 0.24). Because the logarithmic size ratios were positive, this suggests that on average participants reported a *reduction* in the perceived size of the test relative to the reference stimulus for both contexts of the Ebbinghaus illusion; however, this effect was weaker for small than large inducers. In particular, while the illusion effect for large inducers was significantly greater than zero at the group level (mean±SEM: 0.079±0.014, t(25) = 5.78, p<0.001, d = 1.13), the effect for small inducers was not (mean±SEM: 0.01±0.012, t(25) = 0.83, p = 0.412, d = 0.16).

There were also significant differences in the slopes of the psychometric curves for the three illusion configurations (F(2,50) = 25.46, p<0.0001). Specifically, the slopes for both the small inducers (t(25) = −5.19, p<0.0001, d = −1.02) and the large inducers (t(25) = −5.65, p<0.0001, d = −1.11) were significantly smaller than slopes for the control stimulus. The difference in slopes between the two configurations of the illusion only trended towards statistical significance (t(25) = 1.77, p = 0.088, d = 0.35). This suggests that relative to the control stimulus the presence of either illusion configuration interfered with participants' ability to perform the size judgment task, and the ability may also vary for the two illusion contexts.

Illusion strength was measured separately in each visual hemifield for each participant. Even though there was only a significant illusion effect for large inducers at the group-level, there were considerable individual differences in the illusion measurements for both inducer configurations. Closer inspection revealed that while illusion strength for the large inducer configuration was highly variable (−0.15–0.27), it was positive for the majority of participants and hemifields (Figure 2B). In contrast, for the small inducer configuration it was negative for about half of participants and hemifields (−0.14–0.21). This means that even for small inducers there was a strong illusion effect in many individuals; however, because for some individuals the illusion was negative while others showed positive effects, the effects cancelled out in the group average.

While the illusion strength for large inducers was stronger than for small inducers, there was a strong correlation across individuals between the two illusion strengths (ρ = 0.68, p<0.001, 95% conf. int. for ρ: [0.46, 0.82]). In this analysis we treated measurements from left and right visual field as separate measurements. There also was a strong correlation between illusions measured in the left and the right hemifields (ρ = 0.55, p<0.001, [0.33, 0.72]). In this analysis we treated measurements made with large and small inducers as separate measurements. Taken together, these findings suggest that illusion effects for both contexts may be mediated by related processes.

We also tested whether the measurement of illusion strength was related to the bias for the control stimuli. There was a moderate correlation between the bias in size judgments and the illusion strength for small inducers (ρ = 0.30, p = 0.03, [0.01, 0.55]) as well as that for large inducers (ρ = 0.37, p = 0.008, [0.10, 0.60]). This suggests that general biases in size perception may contribute weakly to the measurement of illusion strength. Critically, there was no correlation (Figure 2C) between either illusion strength and size discrimination slopes for control stimuli without any inducers (small: ρ = −0.17, p = 0.235, [−0.42, 0.11]; large: ρ = −0.04, p = 0.78, [−0.33, 0.24]). Therefore, illusion strengths were not confounded by general size discrimination ability.

### Visual cortex area and illusion strength

**Figure 3:**
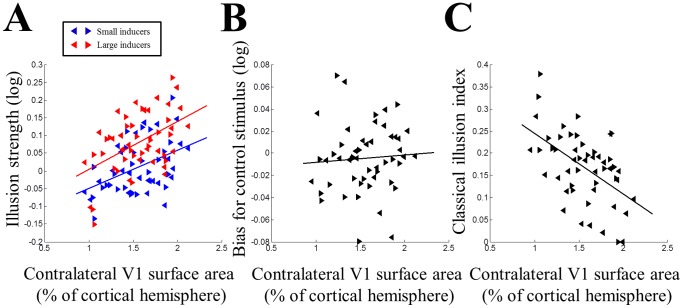
Illusion strength and V1 surface area. A. Individual illusion strengths plotted against the surface area of contralateral V1 (expressed as percentage of the cortical hemisphere). Blue: small inducers. Red: large inducers. B. Bias in size perception for the control stimulus (without any inducers) plotted against contralateral V1 surface area. C. “Classical illusion index” calculated from the illusion strengths for small and large inducers plotted against contralateral V1 surface area. In all plots, left and right pointing triangles denote measurements from left and right visual hemifields in individual participants, respectively. Solid lines are linear regression fits.

We also collected fMRI data and used standard retinotopic mapping [11] to measure the surface area of central V1 and related areas (extending up to 8° eccentricity) in early visual cortex separately for each cortical hemisphere. This confirmed earlier findings of considerable variability in the size of these regions across healthy human participants. To control for any confounding effects of general brain size, we normalized the surface area of each participant's V1 to the overall surface area of the entire cortex. Despite that, the area of V1, expressed as a percentage of whole cortex, was very variable across the sample (0.95–2.12%; see also Figure 3A, *x*-axis). The surface areas of left and right halves of V1 were strongly correlated across participants (ρ = 0.66, p<0.001, [0.32, 0.85]).

Next we sought to test whether reported illusion strength for either context could be predicted by V1 surface area. Unless specified, in this and all following analyses we treated measurements from left and right hemifields as separate and compared each to data from its contralateral cortical hemisphere. Both the illusion for small inducers (ρ = 0.43, p = 0.002, [0.18, 0.64]) and that for large inducers (ρ = 0.41, p = 0.003, [0.13, 0.63]) were correlated with the surface areas of the V1 halves contralateral to the illusion stimulus (Figure 3A). In contrast, the bias for the control stimulus was not correlated with contralateral V1 area (Figure 3B; ρ = 0.14, p = 0.313, [−0.14, 0.40]), although the difference between this correlation and those for either illusion context only approached statistical significance (William's test for comparing two correlations with a common variable; small: t_2_(49) = −1.90, p = 0.064; large: t_2_(49) = −1.79, p = 0.079). The effect remained highly significant also when illusion strengths were corrected by subtracting the bias measured for the control stimuli (small: ρ = 0.4, p = 0.004, [0.14, 0.61]; large: ρ = 0.39, p = 0.005, [0.11, 0.61]). The slopes of linear regressions predicting the two illusion strengths by V1 surface area were also very similar (β_small_ = 0.11; β_large_ = 0.14) and the 95% confidence intervals estimated through 10000 bootstrapping iterations were widely overlapping (small: [0.05, 0.16]; large: [0.06, 0.21]) further supporting the hypothesis that the two contexts are mediated by related mechanisms. The strength of any correlation is bounded by the reliability of the variables. To assess the reliability of our retinotopic definition of V1 we repeated the mapping in a blinded fashion and calculated Cronbach's alpha as a measure of internal consistency (α = 0.82) across all 52 hemispheres. Similar results were observed when analyzing consistency separately for each hemisphere (left: α = 0.72; right: α = 0.86).

We further confirmed the results of these bivariate correlation analyses using a linear regression analysis in which we used the surface areas of left and right V1 as well as the strength of the bias in the control stimuli as regressors to predict the strength of the illusion for either small or large inducers. Both models produced a significant fit (small: R^2^ = 0.31, p = 0.003; large: R^2^ = 0.34, p = 0.001). The beta parameters estimated for how V1 area affects illusions were very consistent, both for the left hemisphere (β_small_ = 0.15; β_large_ = 0.17) and the right hemisphere (β_small_ = 0.08; β_large_ = 0.11). These estimates are similar to the beta parameters from the simple bivariate models. The betas for the control biases were greater than for the surface areas (right hemifield: β_small_ = 1.04; β_large_ = 1.05; left hemifield: β_small_ = 0.22; β_large_ = 0.58), although their confidence intervals were also considerably wider and overlapped zero for the left hemifield. This suggests that they were not as statistically reliable as the influence of V1 surface area on subjective perception, which is consistent with the correlation analyses.

We also tested the correlations between illusion strengths and the *ipsilateral* halves of V1. Because left and right V1 were correlated in size, it is probably unsurprising that these correlations were also significant (small: ρ = 0.35, p = 0.012, [0.07, 0.58]; large: ρ = 0.38, p = 0.006. [0.122, 0.591]). The correlations were however somewhat weaker than for the biologically plausible contralateral hemispheres although this difference was not significant as the confidence intervals for the correlation coefficients were largely overlapping.

We further tested whether these correlations were present only for the surface area of V1 up to a certain eccentricity or if the effect was more general. For example, it is possible that the association between subjective size perception and V1 area was only present for that part of V1 encoding the illusion stimulus but not the regions outside that. Using eccentricity maps (where present, i.e. in 36 hemispheres) we determined iso-eccentricity contours from 0° to 8° in steps of a third degree (corresponding to phase steps of 15°). This showed that the correlation was positive for both large and small inducers at all eccentricities tested but that it was maximal for the largest eccentricity of 8°. An alternative possibility could have been to analyze the correlation separately for bands of V1 defined by a narrow eccentricity range. However, this approach is problematic because the variability of these cortical areal measurements does not scale linearly with the mean of the area, and thus the signal-to-noise ratio of this analysis is strongly reduced.

Since both illusion contexts shared a similar relationship with V1 surface area, it appears that the magnitude of the actual Ebbinghaus illusion, in which both contexts are presented simultaneously, is determined by the *difference* between the two contextual effects. In the most extreme cases, in individuals with large V1 area for whom both contexts produce a strong reduction in perceived size, overall illusion strength will be weak because both targets appear similar. Conversely, for individuals with small V1 the effect for both contexts will be smaller; however, while large inducers still produce a reduction in perceived size, for small inducers the effect will now be of the opposite sign, that is, the illusion manifests as a perceived *increase* in target size.

We can therefore estimate the strength of the classical Ebbinghaus illusion from these data by a linear combination of the two separate effects. However, because both effects were linearly related to V1 surface area with approximately similar slopes, we cannot simply use the difference between the two effect strengths to compare classical illusion strength with V1 area, because calculating the difference will remove the shared variance explained by V1 area in each effect and thus only leave random variance. Instead, we calculated for each participant the mean of both illusion strengths. Then we subtracted these numbers from the maximum of this measure across participants. This “classical illusion index” thus gave a high score to individuals for whom the two illusions were very different (frequently of opposite sign) and therefore far from the maximum of the means, while the individual with the highest mean effect scored zero. (Note that the subtraction from the maximum is an arbitrary scaling factor to ensure that large differences between the two illusion configurations scored highly; we could have chosen any positive number but it would not have any effect on the correlation). Consistent with our earlier reports [7], this estimate of the classical Ebbinghaus strength was also negatively correlated with contralateral V1 area (Figure 3C; ρ = −0.48, p<0.001, [−0.68, −0.22]).

Next, we analyzed the correlations separately for each cortical hemisphere with the illusion in its contralateral visual field. Naturally, these analyses have reduced statistical power because the sample size has been halved. However, even these analyses confirmed qualitatively comparable results to the main analyses. There was a negative correlation between the classical illusion index for stimuli in the left visual field with right V1 surface area ((ρ = −0.57, p = 0.003, [−0.8, −0.21]). There was also a negative, albeit not statistically significant, correlation in the opposite direction between the illusion in the right visual field and left V1 area (ρ = −0.32, p = 0.114, [−0.64, 0.09]). As the confidence intervals of these two correlations are largely overlapping this suggests that there was no hemispheric asymmetry for this brain-behavior correlation, unlike in our previous study [7]. If anything, the asymmetry was the inverse of that we previous observed.

For completeness, we also analyzed the relationship between illusion strengths and the surface areas of neighboring retinotopic visual areas V2 and V3. We observed a correlation between the illusion strength for small inducers and V2 area (ρ = 0.36, p = 0.008, [0.10, 0.58]) but for large inducers this effect did not reach significance (ρ = 0.19, p = 0.172, [−0.09, 0.45]). Neither illusion was correlated with the surface area of V3 (small: ρ = 0.22, p = 0.126 [−0.07, 0.47]; large: ρ = 0.05, p = 0.739, [−0.24, 0.31]). Some shared relationship between early regions is to be expected because earlier work reported a correlation between the surface areas of these visual areas [18], and it is reasonable to assume that if perceived size is represented retinotopically it will be propagated to higher areas in the visual processing hierarchy. Consistent with this we observed a strong correlation between the surface areas of V1 and V2 (ρ = 0.49, p<0.001, [0.23, 0.70]), but not between V1 and V3 (ρ = 0.18, p = 0.202, [−0.10, 0.44]) or between V2 and V3 (ρ = 0.21, p = 0.14, [−0.09, 0.48]). Moreover, partial correlations between V2 area and subjective size perception after removing the variance of V1 area were not significant (small inducers: ρ = 0.14, p = 0.318; large inducers: ρ = −0.07, p = 0.61).

### V1 surface area and size discrimination

**Figure 4:**
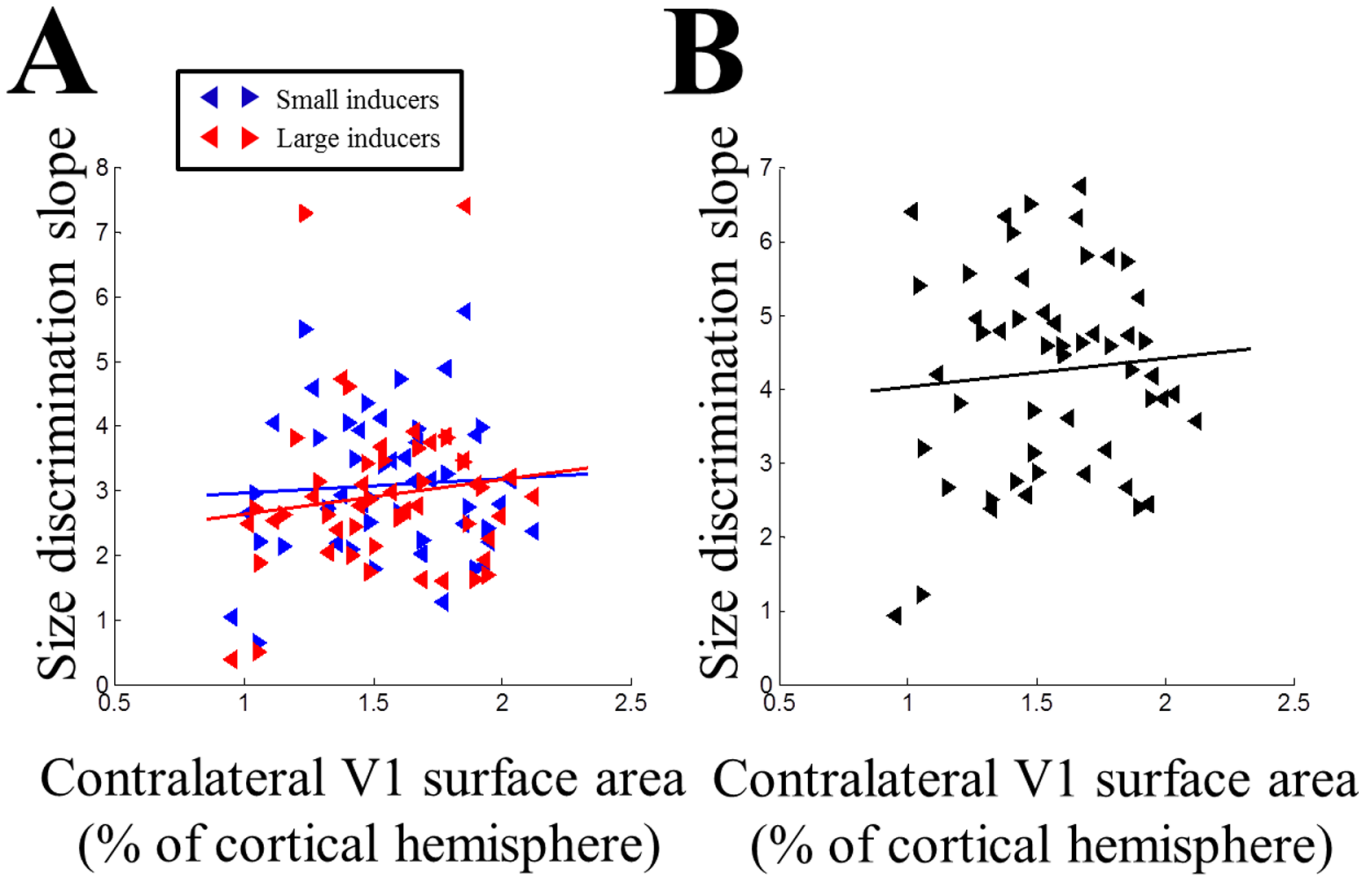
Size discrimination and V1 surface area. A. Individual discrimination slopes plotted against the surface area of contralateral V1 (expressed as percentage of the cortical hemisphere). Blue: small inducers. Red: large inducers. B. Individual discrimination slopes for the control stimulus (without any inducers) plotted against contralateral V1 surface area. In both plots, left and right pointing triangles denote measurements from left and right visual hemifields in individual participants, respectively. Solid lines are linear regression fits.

Next we analyzed the relationship between visual cortical surface area and performance in the illusory size discrimination task (see Methods), using the slopes of the psychometric curves in each participant as a measure of their discrimination sensitivity. This revealed no systematic correlations between V1 surface area and the slopes for either illusion (Figure 4A; small: ρ = −0.01, p = 0.952, [−0.31, 0.28]; large: ρ = 0.11, p = 0.425, [−0.18, 0.39]). Importantly, there was also no correlation between the slopes for the control stimulus without any inducers and V1 surface area (Figure 4B; ρ = −0.02, p = 0.901, [−0.31, 0.27]) and this was significantly different from the correlations with the two illusion strengths (small: t_2_(49) = −2.25, p = 0.029; large: t_2_(49) = −2.23, p = 0.03). This demonstrates that V1 surface area was specifically related only to *illusion strength*, that is, the bias in perceiving different sizes and not to the ability to perform the size discrimination task *per se*.

### Relationship with anatomical V1

**Figure 5:**
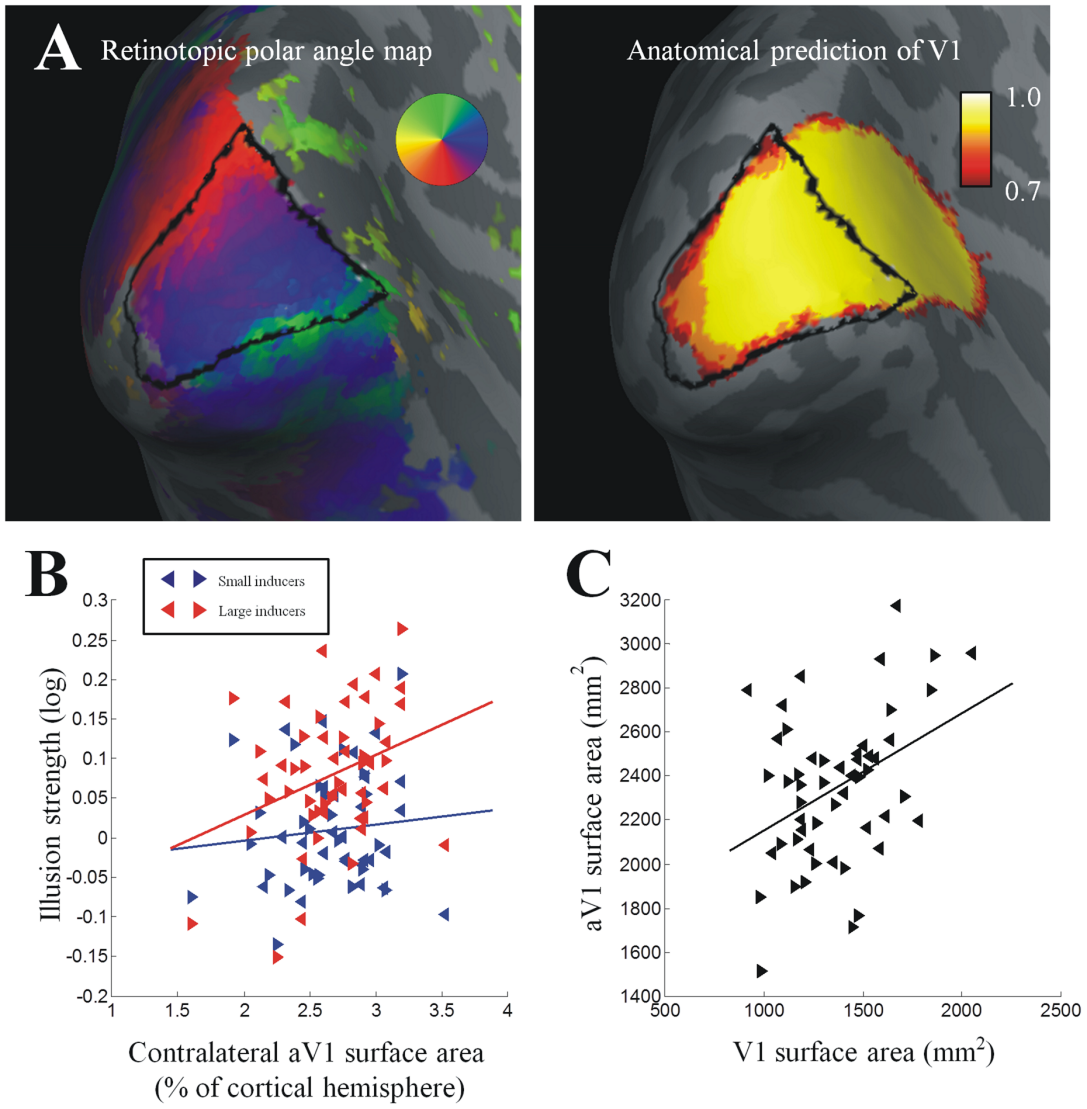
Anatomical definition of V1. A. Left: Retinotopic polar angle maps from a typical participant shown on an inflated reconstruction of the grey-white matter boundary. Shades of gray indicate gyri and sulci. The colour indicate polar angle coordinates of visual field positions mapped onto the cortex. Right: The probability (p>0.7) that occipital vertices fall within aV1 as determined by anatomical criteria [19]. The overlaid black line denotes the boundaries of V1 delineated functionally through retinotopic mapping. B. Individual illusion strengths plotted against the surface area of contralateral aV1 (expressed as percentage of the cortical hemisphere). Blue: small inducers. Red: large inducers. C. Anatomical aV1 surface areas plotted against retinotopic V1 surface areas. In both plots, left and right pointing triangles denote measurements from left and right visual hemifields/cortical hemispheres in individual participants, respectively. Solid lines are linear regression fits.

Our measure of V1 surface area was based on retinotopic mapping using a visual stimulus that extended out to 8° eccentricity. Due to cortical magnification this comprises a large proportion of overall V1 surface area; however, there is still a considerable cortical territory in V1 that our method could not measure. In order to estimate the surface area of the entirety of V1 we employed a probabilistic method implemented in Freesurfer [19]. This procedure predicts the extent of anatomical V1 (aV1) using the cortical folding pattern: surface-based inter-subject alignment is performed to bring cortical landmarks into register. Each vertex is subsequently assigned a probability of being inside V1. This is based on an atlas sample comprising ten brains in which the Stria of Gennari (a micro-structural marker for V1) have been identified *ex vivo*. To be conservative, only vertices on the cortical surface predicted to be within V1 with a probability of greater than 0.7 were included in this region (note that very similar results were observed with different thresholds; however, 0.7 is most comparable to the automatic prediction algorithm implemented in Freesurfer and at much lower thresholds the prediction is frequently biologically implausible). Generally, this prediction was very accurate with substantial overlap of aV1 with our functional delineation of central V1 (Figure 5A). On average the proportion of vertices in the retinotopically defined V1 that were also within aV1 was 0.81±0.01 (mean±SEM). The surface area of aV1 was correlated with the overall surface area of the whole cortical hemisphere (ρ = 0.37, p = 0.007 [0.1, 0.59]). This is unsurprising because its definition is related to an atlas of cortical folding and may thus scale with brain size.

We found no correlation between illusion strength for small inducers and aV1 surface area (Figure 5B; ρ = 0.11, p = 0.436, [−0.21, 0.41]). For large inducers there was a weak positive correlation but the confidence interval overlapped zero (ρ = 0.29, p = 0.035, [0, 0.55]). There was a moderate relationship between the surface area of central V1 defined functionally and aV1 surface area (Figure 5C; ρ = 0.32, p = 0.02, [0.01, 0.60]), although this may have been driven by outliers as the nominal confidence interval for this correlation was [0.05, 0.55]. This is further corroborated by the fact that the more robust Shepherd's *pi* correlation was not significant (π = 0.26, p = 0.141). We also automatically delineated that portion of anatomical aV1 that was significantly activated by our polar mapping (wedge) stimulus. Due to the close correspondence between polar angle borders and cortical folding the variability of this region is therefore mainly driven by the extent of retinotopic activation along the anterior-posterior axis. The surface area of this region was also correlated with the illusion for large inducers (ρ = 0.38, p = 0.005, [0.09, 0.63]). Although the effect for small inducers did not reach significance (ρ = 0.21, p = 0.134, [−0.09, 0.49]), it followed the same trend: this analysis strongly replicated the effect for the classical illusion index combining both stimulus configurations (ρ = −0.46, p<0.001, [−0.67, −0.2]). Together with the good overlap of the anatomical and functional definitions of V1, this suggests that our measure of retinotopically defined central V1 quantifies the degree of *central cortical magnification*, that is, how much cortical territory is allotted to representing the central 8° of visual space (in each hemisphere) rather than how large V1 is in total.

### Gender and age effects

Previous work suggests that Ebbinghaus illusion strength may be weaker in males than females [20]. We did not set out to explicitly test this hypothesis so we did not collect equal amounts of data from males and females. Nevertheless, we also compared the illusion strengths between gender after pooling data for both hemispheres in each participant. This suggested weaker illusion strengths in males (small: t(24) = −2.15, p = 0.042; large: t(24) = −2.42, p = 0.023). To exclude the possibility that this effect could have interfered with the main result, we calculated partial correlations between illusion strengths and contralateral V1 surface area after removing the variance afforded by participant gender. This confirmed a relationship between V1 area and subjective size perception (small: ρ = 0.39, p = 0.005, [0.11, 0.61]; large: ρ = 0.34, p = 0.013, [0.04, 0.58]). We found no gender differences in the slopes of the psychometric curves (small: t(24) = 1.69, p = 0.103; large: t(24) = 0.76, p = 0.458).

We also analyzed whether the age of participants could have confounded the results. Age is a determinant of cortical thickness and may be related to changing neurotransmitter levels [21]. However, we found no correlations between the age of participants and illusion strength (small: ρ = 0.08, p = 0.706, [−0.3, 0.46]; large: ρ = 0.17, p = 0.393, [−0.2, 0.54]) or V1 surface area (ρ = −0.13, p = 0.515, [−0.47, 0.32]). However, we did observe a strong correlation between age and the slopes of the psychometric curves for small inducers (ρ = 0.62, p<0.001, [0.31, 0.85]), while those for large inducers did not reach significance (ρ = 0.33, p = 0.104, [−0.08, 0.66]). This may be indicative of changes in the ability to make the fine visual discriminations in this task although it was not robust over both illusion contexts. More importantly, it demonstrates that illusion *strength* was not confounded by the age of participants.

## Discussion

Here we tested individual differences in the strength of the Ebbinghaus illusion resulting from two different inducer contexts; that is, target stimuli surrounded by small or large inducers, respectively. We observed considerable variability in illusion strength for both contexts. Importantly, our findings are also consistent with our earlier report [7] that inter-individual variability in both illusion strengths is strongly correlated with the surface area of central V1. When considering the average illusion strength across all participants, we only found a significant effect for large inducers. This was due to the fact that for small inducers the sign of illusion strengths (whether it afforded an increase or decrease in perceived size) was opposite in roughly half of participants and so the effect canceled out in the group average. This fact underlines the importance of studying inter-individual variability rather than focusing only on the mean across a population.

Our results help to resolve several important questions about these earlier results. First, they suggest that the effects for both illusion configurations are mediated by related mechanisms, potentially local circuits in V1. When the target was surrounded by large inducers most participants experienced the target to be reduced in size. However, when the target was surrounded by small inducers, many participants perceived an increase in size while others still perceived a (lesser) reduction. The magnitudes of these two effects were also strongly correlated across individuals. The classical Ebbinghaus illusion is therefore characterized by the *difference* in perceived size of the two targets. When the effect for both configurations is strong, the difference is small and therefore the classical illusion is weak. When the two effects are very different, the classical illusion is strong.

Our present findings also did not replicate the hemispheric asymmetry we observed in previous work [7]. Illusion strength was measured separately for each visual hemifield and correlated with the surface area of contralateral halves of V1. Thus combining the data from both separate hemispheres substantially enhanced statistical power. However, even analyzing the correlation separately for each hemisphere/hemifield pair should have had sufficient power to reveal a strong asymmetry as in our previous study. While the correlation between left visual field illusion and right V1 area did not reach statistical significance in our present study, it was not different in sign from the corresponding correlation between right visual field illusions and left V1 area. More importantly, even if this asymmetry were a real effect it would be the opposite of our previous findings. There was also good internal consistency of our retinotopic measurement of V1 surface area on a repeated delineation of the areas. If anything, the reliability of right hemisphere measurements was greater than for the left hemispheres.

This may suggest that the hemispheric asymmetry we reported previously may have been artifactual rather than reflecting a genuine lateralization of size judgments in early visual cortex. Variability in our measurement of right V1 in our earlier study may have masked the relationship. Another possibility is that the way we measured illusion strength in that study, by presenting the two configurations simultaneously to the left and right hemifields, may have resulted in participants judging the illusion based on only one hemifield, reflecting natural biases in attentional deployment. For example, the stimulus in the right visual field could have been the only relevant one: participants could fail to accurately compare the sizes of the two stimuli but rather compare the right stimulus to a mental representation of “average size”. This problem would have been particularly severe for the smallest size differences near the point of subjective equality, which would have interfered with the estimation of illusion strength. In our present experiments, participants were forced to judge the illusion in one hemifield while the unchanging reference without inducers (and thus without any illusion) was shown to the opposite hemifield. This means that only one hemifield could directly contribute to *subjective* perception. Even if participants had selectively only judged the stimulus on the right, by ignoring the illusion stimulus on the left they should have made only veridical (minimal bias) size judgments, which we did not observe. Moreover, it is likely that by only presenting one illusion stimulus in each trial next to a simple reference, participants' attention was drawn to the side where the illusion was presented thus helping with the comparison.

Here we also measured participants' sensitivity to judging the size of two stimuli in the absence of any inducers generating an illusion. This allowed us to test whether variability in subjective illusion strength could be trivially explained by differences in the objective ability to make fine spatial discriminations. We found no relationship between their discrimination ability (slopes of psychometric curves) and the strength of either illusion configuration. Moreover, there was no significant correlation between discrimination ability and V1 surface area and this relationship was significantly weaker than those between V1 and the illusion strengths. Even though previous research reveals that another form of fine spatial discrimination, Vernier acuity [10], is correlated with local V1 area, judging the size of two simple visual stimuli is not related to V1. It also indicates that size discrimination ability is not likely to be a limiting factor in making judgments on the illusory size differences. We did however observe small *biases* in size judgments even for control stimuli without inducers (e.g. two identical stimuli might appear to a participant as being unequal). These biases were related to illusion strength, which is not surprising because these biases in size judgment may of course contribute to judging the size of the illusion stimuli. However, our critical results were robust even when illusion strength was corrected by subtracting these small biases.

Finally, our previous work defined visual areas only functionally with retinotopic mapping and measured the surface area of only the stimulated portion of V1 representing the central visual field. Here, we also employed a probabilistic procedure predicting the full extent of V1 based on anatomical landmarks [19]. This revealed that the area of the whole of V1 defined by these anatomical criteria was unrelated to illusion strength. There was, however, an excellent correspondence between the anatomical prediction and the retinotopic definition of V1. The factor causing this discrepancy must therefore lie in how large a proportion of the anatomical V1 is encompassed by retinotopic V1 – the amount of central cortical magnification. Therefore, a significant part of the variance in illusion strength is explained by individual differences in the extent to which an individual's V1 selectively represents the central visual field. Interestingly, the Ebbinghaus illusion may be in part based on hereditary factors [22] which could further allude to its relationship with cortical morphology.

### Potential neural mechanisms

What neuronal circuits could give rise to the Ebbinghaus illusion? The motivation for our original experiments comparing Ebbinghaus strength to V1 area [7] was to test whether the effect of these circuits is inversely related to cortical distance. In a larger cortical area the distance between the target stimulus and inducers will be larger. Therefore, if the illusion is mediated by local circuits and dependent on cortical distance – either because signals are slow to propagate or weaken with distance – the illusion effect must be reduced when cortical distances are greater.

Our present findings are consistent with that hypothesis. However, interestingly we found a discrepancy in the strength and the sign of the illusion effect for small inducers. While for most participants the effect of large inducers was to reduce the perceived size of the target, for small inducers the effect varied considerably with a considerable number of participants also showing a (somewhat counterintuitive) reduction in perceived size for this stimulus configuration. Importantly, because both these effects were correlated with V1 area there appears to be a critical V1 surface area at which this effect changes from being an increase to a decrease in perceived size.

We did not aim to test the effect of inducer size *per se* but our stimulus parameters were chosen based on pilot work to produce reliable and strong illusion effects. Therefore, the two stimulus configurations not only manipulated inducer size but also the target-inducer distance in visual space: for large inducers the distance was considerably greater than for small inducers. Under these circumstances the Ebbinghaus effect is probably related to another contextual modulation of size perception, the Delboeuf illusion. Only when the target-inducer distance is very small *and* the surround is largely covered by inducers (as in our experiment) there is an increase in perceived size, which replicates previous results [9]. In fact, predicting the mean illusion strength for our stimuli by extrapolating from the results of Roberts et al. [9] produces fairly similar results (small inducers: 0.01–0.02; large inducers: 0.11) to our observed illusion strengths (small: 0.01; large: 0.08). Based on their results we would not expect any drastic differences for a wide range of stimulus parameters, and thus are confident that our findings are not only specific to the stimuli we used. Using the beta parameters estimated from the linear regression analysis we can predict that a change in V1 surface area of 1% of the overall cortical area would result in a change of illusion strength of ∼0.13 (averaged across hemispheres and inducer configurations). Relative to someone who experienced no illusion at all, an individual with this illusion strength would perceive the stimulus with large inducers as 88% of its true size.

**Figure 6:**
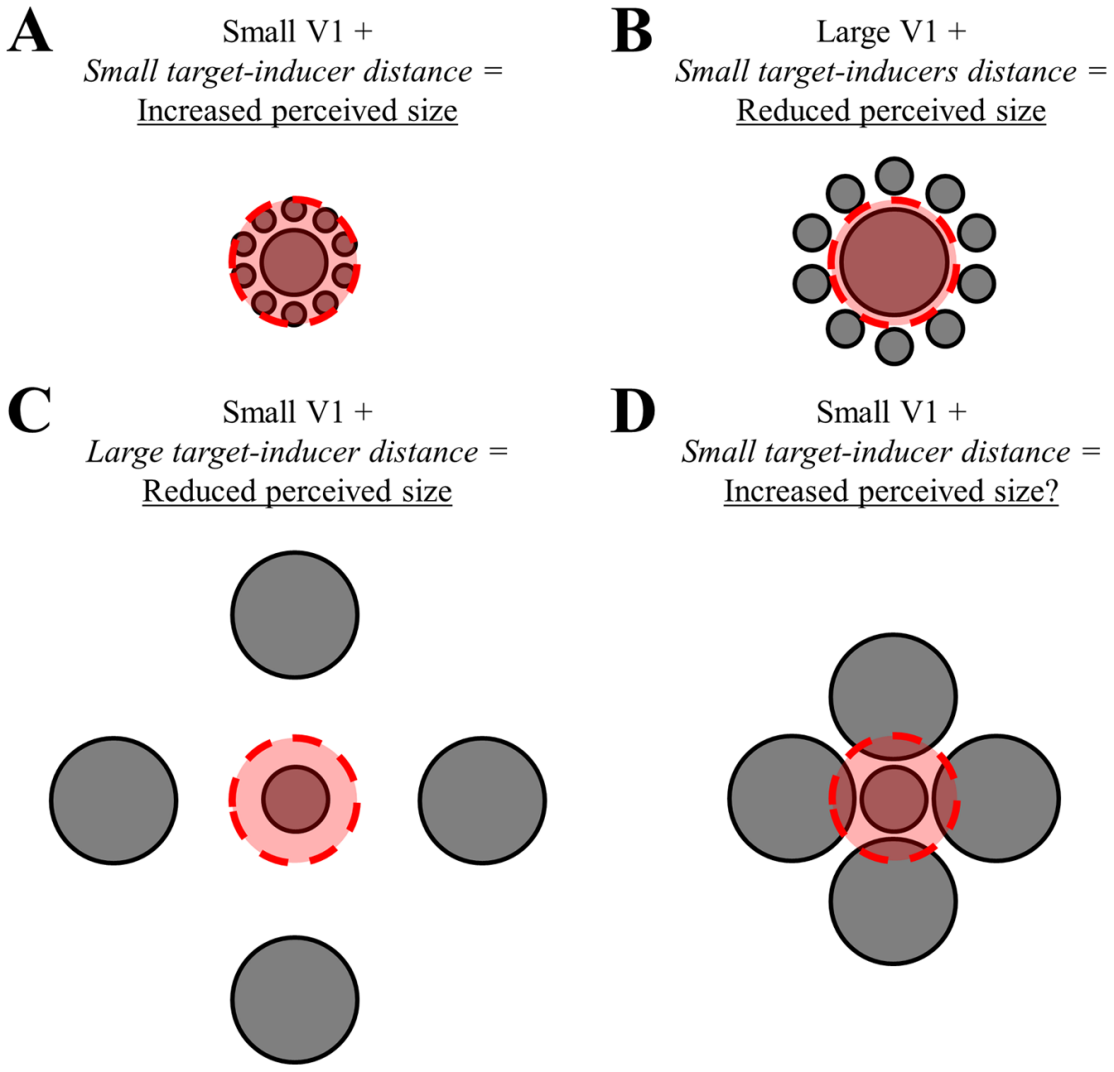
Schematic illustration of the relationship between cortical representation of the target-inducers distance and illusion strength. A. In a small V1, the target-inducer distance is small and thus falls within a constant range (dashed and shaded red circle). This affords a perceived size increase of the target. B. In a large V1, the target-inducer distance is greater than the constant range. This affords a perceived size reduction of the target. C. For large inducers, even in a small V1 the target-inducer distance is large and thus falls outside the constant range. In all situations this results in a perceived size reduction. D. Theoretically, if large inducers were positioned such that the target-inducer distance falls within the constant range, a size increase should be perceived. Some readers may in fact confirm this effect by visual inspection of this image.

Our results suggest that the critical distance (Figure 6, red circles) for the direction of the illusion effect is relative to cortical architecture rather than visual space: in a small V1 the cortical distance between target and the small inducers is small enough to afford a perceived size increase (Figure 6A). In larger V1s the retinotopic representation of the stimuli is scaled up such that the inducer falls out of this critical range and the effect is a perceived size reduction (Figure 6B). Due to our stimulus design, the distance of large inducers was always such that it would reduce perceived size (Figure 6C) – however, our findings suggest that if the cortical distances between large inducers and targets were small (i.e. in a small cortex and at small distance in visual space) they, too, may produce a perceived size *increase* (Figure 6D). In fact, some readers may subjectively confirm this prediction by comparing the perceived sizes for the targets in Figures 6C and 6D.

Future work on size perception and V1 area should therefore control these factors by using the Delboeuf [23] rather than the Ebbinghaus illusion and manipulating the distance and the size of the inducer separately. Moreover, by constructing Ebbinghaus stimuli that fix these parameters (see e.g. [24]) it will be possible to disentangle the effects of size contrast and local contextual effects of proximity and inducer size. We believe that our results speak to local interactions between adjacent stimuli that are mediated by circuits within V1. An interesting possibility to test this hypothesis further would be to measure illusion strength across a range of different eccentricities; as cortical magnification decreases the illusion should become stronger. However, this is complicated by the interference from crowding effects with the measurement of the illusion in the peripheral visual field. Moreover, it is remains unknown whether these contextual interactions observed in the central visual field are also present in the periphery.

Note however that it is also conceivable that size contrast is a higher-level process independent of V1. Consistent with this, previous work has also suggested that the Ebbinghaus illusion may depend on complex stimulus characteristics, such as the figural or conceptual similarity between targets and inducers [25]–[27] and prior knowledge of object size [28]. While it is difficult to completely rule out low-level stimulus effects in these experiments [29], [30], they suggest that top-down processes also contribute to the Ebbinghaus illusion. This is further supported by the fact that even our strongest brain-perception correlation, the relationship between V1 area and the strength of small inducers, accounts only for ∼18% of the variance. This is not a weak effect, as correlations explaining more than 25% variance are highly unlikely for noisy measures based on functional neuroimaging and cortical architecture [31]. Nevertheless, this suggests that factors other than V1 area are likely to be involved as well.

### Structural and functional variability in visual cortex

Previous research on individual differences in the physiological functions and neural architecture of early visual cortex implicates a number of processes that may mediate the effects we describe here. The peak frequency of visually induced gamma oscillations measured in early human visual cortex with magnetoencephalography (MEG) correlates with the concentration of the inhibitory neurotransmitter GABA [32] and with the ability to make fine orientation discriminations [33]. We recently reported that retinotopically defined V1 surface area correlates with peak gamma frequency [34]. We interpreted this as evidence for more homogeneous local networks in larger V1; that is, greater cortical magnification. This is consistent with recent reports that gamma frequency decreases with stimulus eccentricity [35]. In addition, this could also indicate greater inhibitory drive in larger cortices that manifests as a reduction in perceived target size for all but the smallest target-inducer distances.

The causal role of inhibitory processing, particularly that mediated through GABA-ergic circuits, could be investigated pharmacologically in future studies. Moreover, there is evidence that gamma frequency (and presumably GABA concentration) decreases with age [21]. This could mean that the illusion strength as measured here will in fact increase with age. This is also consistent with reports that children do not experience the Ebbinghaus illusion [36], which could be related to greater inhibitory activity at a younger age, although this may be trivially explained by technical problems measuring illusion strength in children [37]. We did not find an age-dependence in our data but our sample deliberately only included young adults for whom such an effect would be subtle.

One limitation of our measurement is the use of traditional phase-encoded retinotopic mapping techniques [11]. These methods are robust for analyzing polar angle maps in limited and noisy data and thus sufficient for delineating early visual regions reliably. However, they do not provide reliable information about population receptive fields (pRF), that is, the spatial spread of retinotopic activation, and they are likely to be biased in the eccentricity dimension [17]. Using pRF mapping analysis based on forward models [17], [38] or data-driven methods [39], [40] will provide more accurate maps and a wealth of additional information about the functional architecture of visual cortex. For instance, pRF models with center-surround antagonism where an inhibitory surround modulates responses from a central excitatory subregion [41] may also help explore the relationship between perception and intra-cortical inhibition. The stimulus design in our present study was not optimized for pRF analysis because of the lack of blank epochs for measuring the baseline response and empirical measurements of the hemodynamic response function. However, we have adapted pRF mapping procedures for future studies. In independent experiments, we are currently determining the optimal experimental and analytical parameters for running such studies in the context of individual difference studies and compare the biases introduced by different analysis pipelines.

One previous study used pRF mapping to investigate the size of the point image, the theoretical region of cortex activated by a visual point stimulus [38]. They observed that while cortical magnification and pRF size are highly variable across the cortex and between different individuals, the point image is remarkably constant. Similar effects have also been reported from neurophysiological measurements in animal models [42]. This may be one indication of the processes we postulate here: constancy in the spatial extent of cortical responses regardless of the overall size of visual cortex is consistent with weaker contextual interactions in larger V1. It is possible that the point image describes the precise range at which we observe perceived size increases (i.e. in individuals with small V1 for inducers close to the target). We used previous measurements of average cortical magnification [10] to estimate the cortical distance between the edges of target and small inducers. This results in a distance of ∼3.3 mm, well within the 3–4 mm range suggested by previous fMRI estimates of the population point image in human V1 [38]. The question whether the critical cortical distance is determined by the lateral extent of horizontal connections within V1, the dispersion of feed-forward and feedback connections between areas or dependent on altogether different factors remains unresolved.

In addition, probabilistic methods for delineating visual cortical regions will probably become useful for validating the measurement of V1 surface area and further characterize the sources of variability. One method to determine not only the spatial extent but the retinotopic organization of V1 based on anatomical morphology and a retinotopic mapping template has recently been presented [43]. It remains unclear how well such methods can capture the inter-individual variability in visual cortical organization that is of interest in studies like ours. Our present results suggest that while the polar angle boundaries of V1 may be well predicted by probabilistic methods, the eccentricity dimension may be associated with greater errors in such procedures. It is this prediction error in local cortical magnification that should correlate with subjective size perception. Certainly independent estimates of the size and cortical magnification in visual regions will help refine our understanding of the link between neural architecture and perception.

### Conclusions

Here we expanded upon our earlier reports that V1 surface area predicts Ebbinghaus illusion strength by showing that this effect is similar for both contexts of the Ebbinghaus illusion (large vs small inducers), that it is independent of general size discrimination ability, that there is no consistent hemispheric asymmetry, and that it is dependent on central cortical magnification rather than the overall anatomical size of V1. We suggest that the illusion is in part mediated by local circuits in V1 that generally afford a reduction in the perceived size of a target stimulus except at very short distances between targets and inducers where the effect produces an increase in perceived size. Future studies should further dissect the component factors of the illusion (e.g. size contrast vs local interactions) and apply advanced retinotopic analysis techniques to disentangle the physiological processes underlying this effect.
